# Modern Conservative Management Strategies for Female Stress Urinary Incontinence: A Systematic Review

**DOI:** 10.3390/jcm14103268

**Published:** 2025-05-08

**Authors:** Aida Petca, Andreea Fotă, Răzvan-Cosmin Petca, Ioana Cristina Rotar

**Affiliations:** 1 Department of Obstetrics and Gynecology, “Carol Davila” University of Medicine and Pharmacy, 8 Eroii Sanitari Blvd., 050474 Bucharest, Romania; aida.petca@umfcd.ro; 2 Department of Obstetrics and Gynecology, Elias University Emergency Hospital, 17 Mărăști Blvd., 050474 Bucharest, Romania; 3 Department of Urology, “Carol Davila” University of Medicine and Pharmacy, 8 Eroii Sanitari Blvd., 050474 Bucharest, Romania; 4 Department of Urology, “Prof. Dr. Th. Burghele” Clinical Hospital, 20 Panduri Str., 050659 Bucharest, Romania; 5 Obstetrics and Gynecology I, Mother and Child Department, “Iuliu Hatieganu” University of Medicine and Pharmacy, 400012 Cluj-Napoca, Romania; cristina.rotar@umfcluj.ro; 6 Obstetrics and Gynecology I Clinic, Emergency County Hospital, 400006 Cluj-Napoca, Romania

**Keywords:** stress urinary incontinence, female, platelet-rich plasma, laser, radiofrequency, bulking agents, stem cell

## Abstract

Stress urinary incontinence (SUI) is characterized by the involuntary leakage of urine during activities that increase intra-abdominal pressure. The management of SUI encompasses surgical treatments, such as colposuspension and sling procedures, and nonsurgical ones that involve pelvic floor muscle treatment, behavioral therapies, as well as pharmacological interventions. By exploring nonsurgical options initially, individuals have the opportunity to address the root causes of stress urinary incontinence and strengthen pelvic floor muscles. **Background/Objectives**: This article delves into the conservative measures in managing SUI among women and the options of minimally invasive strategies for SUI, such as the injection of platelet-rich plasma, stem cells, bulking agents, and laser and radiofrequency therapy. **Methods**: A search of the literature from 2010 until January 2024 was carried out on PubMed, Cochrane Library, and Web of Science research databases. **Results**: A total of 34 studies on human females assessing the roles of platelet-rich plasma, laser and radiofrequency therapy, bulking agents, and stem cell therapy were included. **Conclusions**: The shortcoming of most conservative techniques seems to be represented by the temporary effects and the necessity of repeated treatments. To establish effective medical techniques, adopting more standardized procedures and conducting comprehensive randomized controlled trials is imperative.

## 1. Introduction

Stress urinary incontinence (SUI) is a prevalent condition, particularly among women, characterized by the unintentional leakage of urine during activities that increase intra-abdominal pressure, such as coughing, sneezing, laughing, or exercising [1]. The quality of life, self-esteem, and social engagement of women may be adversely affected by stress urinary incontinence [2].

In recent decades, there has been a remarkable increase in life expectancy globally, coupled with a shifting trend in the age at which women are choosing to embrace motherhood. This evolving landscape brings forth a number of challenges and opportunities, reshaping societal perceptions and healthcare paradigms. Advancements in healthcare, improved living conditions, and breakthroughs in medical science have collectively contributed to a significant increase in life expectancy. People are living longer, healthier lives, prompting individuals to reconsider the timing of major life events, including starting a family. In 2021, some European Union (EU) member states (Spain, Italy, Luxembourg, and Greece) had mothers giving birth for the first time at an age exceeding 31, an increase of about +1–2 years compared to 2011. Conversely, two EU states (namely, Romania and Bulgaria) had women having their first child at an age below 27, also with an increase in +1–1.5 years compared to 10 years before [3,4].

While surgery can provide relief for stress urinary incontinence, it is essential to acknowledge that it might offer a solution for a limited duration. Over time, the effectiveness of surgical interventions can diminish, and individuals may find themselves facing a recurrence of symptoms. This potential need for additional surgeries underscores the importance of carefully considering and, when appropriate, delaying surgery in favor of conservative measures. Although mid-urethral tapes and colposuspensions are established procedures with demonstrated long-term effectiveness and a success rate of around 80% [5], they carry the potential risks of tape-related complications and the development of pelvic organ prolapse, respectively, as well as lacking a definitive causal therapy. At 5 years follow-up, only 61% of the patients who had undergone surgical mesh implantation reported being satisfied or very satisfied with their quality of life. The primary reason for dissatisfaction was the development of sexual dysfunction, including issues like dyspareunia or incontinence during intercourse [6].

Moreover, complications of mesh implantation could be linked to the materials used, such as mesh exposure or extrusion, shrinkage of the tissue that surrounds the mesh, urinary symptoms, chronic pain, infections, and many others [7]. Understanding crucial mesh parameters, such as porosity, raw material and combination of raw material, weight, elasticity and tensile strength, is essential for selecting the most appropriate mesh [8].

By exploring nonsurgical options initially, individuals have the opportunity to address the root causes of stress urinary incontinence and strengthen pelvic floor muscles. This proactive approach offers relief from immediate symptoms and may contribute to long-term benefits, potentially reducing the likelihood of recurrent issues. Delaying surgery allows women to navigate through the challenges of perimenopause and menopause with a more gradual and adaptive strategy. It provides a window for implementing lifestyle changes and therapeutic interventions that can contribute to sustained improvements in urinary control. This patient-centered approach considers the potential need for future interventions and aims to optimize overall well-being over an extended period.

Given the potential benefits of conservative management and the uncertainties surrounding its long-term impact, a systematic review is warranted. This review will critically evaluate existing evidence on the effectiveness of nonsurgical interventions for SUl, assess their role in delaying or preventing surgery, and explore their impact on long-term urinary control. The findings will provide valuable insights for clinicians and patients, supporting informed decision making and optimizing treatment pathways for women with SUl.

## 2. Materials and Methods

The study protocol was registered on the PROSPERO database of systematic reviews (protocol number CRD420251011505). The 2020 Preferred Reporting Items for Systematic Reviews and Meta-Analyses were followed (see Supplementary Materials). This paper is a systematic review of the specialty literature published in English between 2010 and 2024. A total of 34 studies were included, identified by searching PubMed, Clarivate Web of Science, and Cochrane Library research databases for studies on female humans, assessing the roles of platelet-rich plasma, laser and radiofrequency therapy, bulking agents, and stem cell therapy.

### 2.1. Eligibility Criteria

We included randomized controlled and non-randomized clinical trials on adult female individuals with diagnosed stress urinary incontinence or stress-predominant mixed urinary incontinence, which evaluated the subjective and objective efficacy of the aforementioned therapies for the treatment of surgically naïve patients.

### 2.2. Information Source

A systematic literature search was performed using the PubMed, Clarivate Web of Science, and Cochrane Library databases (last search date: 20 January 2024).

### 2.3. Search Strategy

The identification of these studies involved utilizing combinations of specific keywords, including “platelet-rich plasma”, “bulking agents”, “laser”, “stem cell”, “stress urinary incontinence”, and “female”, as shown in Table 1.

### 2.4. Data Collection

Structured tables were used to extract essential information for all eligible studies. The extracted data included authors’ names, publication year, location, time frame, type of study, number of included individuals, age, therapeutic agents, measured efficacy outcomes, and their subsequent results (including subjective and objective cure rates, as well as cure, improvement and failure rates), and the duration of follow-up.

### 2.5. Data Items

Subjective outcomes were evaluated using self-reported symptoms as well as validated questionnaires. Objective outcomes were evaluated using stress tests, pad tests, voiding diaries, ultrasonography, and urodynamic studies.

### 2.6. Selection Process

Studies were screened by one author and checked by two other authors.

### 2.7. Data Collection Process

Data were extracted by one author and checked by two other authors.

### 2.8. Risk of Bias

The risk of bias was assessed using Cochrane Rob-2 and ROBINS-I tools for randomized controlled trials and non-randomized trials, respectively. The data were reported in Tables S1 and S2 (see Supplementary Materials). Bias assessment was reported using Rob-2 domain.

## 3. Results

**Figure 1:**
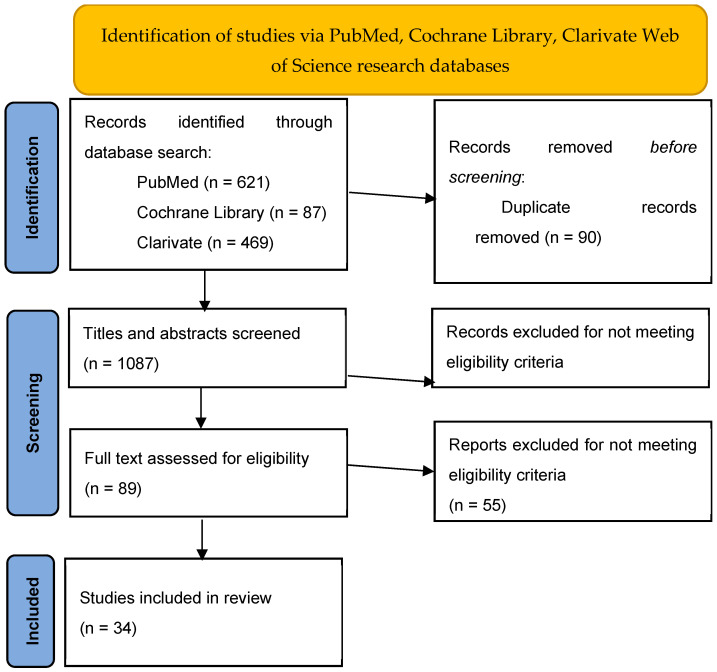
Flow diagram of study identification for the systematic review on conservative treatments for stress urinary incontinence.

We identified a total of 1177 studies up to 20 January 2024. After the exclusion of 90 publications (duplicates), 1087 were selected for screening. At the end of the selection process, 34 studies were included for analysis. A detailed process is reported in Figure 1.

Table 2 includes all the relevant studies selected for analysis and their respective characteristics.

Risk of bias was evaluated across included randomized controlled trials using the RoB-2 tool, assessing five domains of potential bias (see Supplementary Materials—Table S1). The majority of studies demonstrated a low risk of bias across all domains. However, one study (Saraluck [9]) was rated as having a high overall risk of bias due to concerns with the randomization process (Domain 1), raising the potential for selection bias and reduced internal validity. Additionally, several studies (Alexander [16], Gambacciani [24], and Sokol [27]) were judged to have “some concerns” in one or more domains, including missing outcome data and selective reporting. These moderate risks may introduce uncertainty into the findings and should be considered when interpreting the overall body of evidence.

For non-randomized trials, the risk of bias was assessed using a structured domain-based tool evaluating seven potential sources of bias (see Supplementary Materials—Table S2), including confounding, intervention classification, participant selection, deviations from intended interventions, missing data, outcome measurement, and selective reporting. Among the studies included, the majority was rated as having a moderate overall risk of bias, primarily due to concerns in individual domains such as confounding (D1) and participant selection (D3).

Only one study (Fistonic [23]) was judged to have a serious risk of bias, with moderate concerns identified across multiple domains (D1, D3, D5), raising concerns about the internal validity of its findings. Several studies (e.g., Long [10], Athanasiou [11], Brosche [30], Ghoniem [28]) also exhibited moderate risk across multiple domains, particularly in confounding and selection-related biases, which may limit the interpretability of their effect estimates.

Conversely, a substantial subset of studies demonstrated consistently low risk of bias across all domains (e.g., Behnia-Willison [13], Gaspar [17], Sharifiaghdas [39], and Stangel-Wojcikiewicz [41]), reinforcing the reliability of their findings within the broader evidence base.

While the presence of some methodological limitations in a subset of studies may introduce a degree of uncertainty, the overall pattern of findings across studies with low and moderate risk suggests a degree of consistency in outcomes.

### 3.1. Platelet-Rich Plasma Therapy

A-PRP is derived from platelets, evaluated for their potential influence on tissue regeneration, and obtained from the individuals’ blood after centrifugation. These platelets contain a considerable amount of growth factors and cytokines, such as the insulin-like growth factor, epidermal growth factor, basic fibroblast growth factor, platelet-derived growth factor, transforming growth factor-beta, vascular endothelial growth factor, connective tissue growth factor, hepatocyte growth factor, and interleukin 8. Platelets release these growth factors and chemical mediators to support various physiological processes [43,44].

Five studies (see Supplementary Materials—Table S3), out of which two were randomized clinical trials, discussed the role of PRP alone or in combination with either pelvic floor muscle training or CO_2_ laser. In three of the studies, Saraluck [9], Athanasiou [11], and Behnia [13] reported an improvement rate of 60–90% of the studied population. The other two studies, while also indicating statistically significant improvement rates, exhibited comparatively lower subjective and objective rates. The limitations of this comparison included variations in the procedure, such as the injection site and the time interval between administrations. This underscores the need for future studies to establish a standardized procedure for interventions of this nature.

Saraluck et al. [9] ran a randomized controlled trial where statistically significant differences in objective and subjective measurements were found between the a-PRP + PFMT group, compared to PFMT alone, with vastly improved quality of life: 90% of the patients in the combined group experienced a >50% improvement, compared to 14% of those in the PFMT group.

In combination with a CO_2_ laser, PRP should reduce the level of inflammation in the laser-treated areas, by hydrating and optimizing the tissues, activating stem cells, migration and proliferation, therefore promoting healing. Behnia-Willison [13] showed improvement at 3 months after combined treatment, which persisted through the 12–24-month follow-up period. They proved a 92% decrease in surgery necessity, as well as 43% reduced rates of urodynamic studies, which pose cost benefits for both the patient and the national health system.

Grigoriadis [12] compared PRP with a sham group, using sodium chloride 0.9%. Subjective and objective cure rates were significantly higher in the PRP group at 6 months, but with a subjective cure rate of only 32%. Athanasiou [11], using the same interval between PRP injections, documented that, at the 6-month follow-up, 80% of the individuals reported to be at least improved.

Long et al. [10] reported the 60% effectiveness of a single A-PRP injection at both 1 and 6 months, as well as a higher success rate in patients younger than 40 years old, although the latter did not reach statistical significance.

The majority of studies investigating platelet-rich plasma (PRP) utilized RegenLab kits, which provide a platelet concentration of 1.6×. As a result, this concentration has become the standard reference in the literature.

A total of five studies, encompassing 212 patients, have examined the efficacy of PRP. Among these, two studies [10,11], including 40 patients, specifically focused on PRP alone and demonstrated significant and lasting clinical improvements. Additionally, two randomized controlled trials (RCTs) [9,12], involving a total of 110 patients, reported significantly enhanced outcomes when PRP was incorporated into treatment protocols.

### 3.2. Laser, Infrared, and Radiofrequency

Non-invasive laser, infrared (IR), and radiofrequency (RF) therapy operates on the principle of photothermal treatment of connective tissue, causing shrinkage of collagen without destruction, a mechanical pull of deeper tissues, as well as formation of new collagen fibers. Moreover, neocollagenesis, neoangiogenesis, elastogenesis, and increased fibroblast activity occur in the heated area [45]. The key differences between the three consist of wavelength—infrared and radiofrequency have longer wavelengths than laser light; and coherence—laser light is coherent, meaning the waves are in phase and have a narrower beam, whereas the other two are typically not coherent.

From a total of thirteen studies (see Supplementary Materials—Table S4), CO_2_ laser was discussed in five randomized trials, compared to sham treatment in three of these, platelet-rich plasma in one (as discussed in the previous chapter), and radiofrequency and sham treatment in another. They employed different intervention protocols, ranging from three to four times of laser application, 28 to 35 days apart.

Lauterbach [18] compared CO_2_ laser with sham and reported that despite statistically significant differences at 3 months, the follow-up at 6 months showed similar results between groups, aligning with the results of previous studies. Temporary effectiveness of the CO_2_ laser treatment was also supported by Alexander et al. [16], who reported that the sham and active treatment groups had comparable outcomes and health-related quality of life measurements at 3 months. Moreover, the complications of recurrent CO_2_ laser therapy may include chronic vaginal and cervical irritation, leading to discharges, affecting sexual function, and increasing the susceptibility to malignancy and infection with human papillomavirus, as said by Lauterbach [18]. In Temtanakitpaisan’s study [14], the fractional CO_2_ laser treatment did not demonstrate any advantages over the sham technique in relieving symptoms of stress urinary incontinence. The observed enhancement in SUI symptoms could potentially be attributed to pelvic floor muscle training. There were no alterations in bladder neck descent or levator hiatal area immediately after the intervention or three months after the completion of treatment in either group.

Photobiomodulation using laser irradiation was suggested as a treatment of fatigue of the pelvic muscles in a randomized control trial with sham laser, reported by da Silva et al. [22], which used manometric feedback and reported an increase in muscular endurance and strength. These findings highlight the potential of adjunctive photobiomodulation therapy to enhance the efficacy of conventional rehabilitation approaches. The observed improvements may be attributable to infrared-induced enhancements in microcirculation and muscle tissue oxygenation, facilitating greater gains in muscular performance. Nevertheless, the intergroup assessment did not reveal any notable differences.

Radiofrequency effects were discussed in two studies encompassing 250 patients. The effect of transurethral collagen denaturation by radiofrequency was studied by Elser [26] on 136 patients, who reported that, at 18 months post-procedure, 46.7% of the patients in the intention-to-treat population and 61.7% of the patients evaluated reported a reduction in >50% in leaks and significant quality of life improvement. Half of the patients (50.4%) reported to improve to different degrees from baseline. The results are consistent with those reported at 12-month follow-up. As Elser [26] reports, most of the patients who were lost to follow-up had improvements in SUI episodes, UDI-6 scores, and QoL improvement, suggesting that discontinuation might have taken place due to experiencing positive outcomes and opting to forego the additional time commitment.

Another randomized clinical trial conducted by Seki et al. [25] compared radiofrequency to CO_2_ laser treatment and sham laser and found significantly improved yet similar objective cure rates and subjective results between the laser and RF group, as opposed to the sham control group. However, in the sham control group, adherence to behavioral therapy and perineal exercises led to improvement rates that persisted up to 4 months after the first intervention, but this effect was not sustained further.

Seven studies, encompassing 394 patients, discussed the effect of erbium-doped yttrium-aluminum-garnet laser treatment (Er:YAG). They reported good long-term results, comparable to those of surgical interventions; two [17,20] of the studies discussed the importance of maintenance interventions at 6 months, with both of them initiating the treatment with two sessions of Er:YAG laser treatment, however, at 3, respectively, 4 weeks apart. Among these studies, Er:YAG laser treatment alone was investigated in five studies, encompassing a total of 343 patients. While the results demonstrated significant clinical improvement, they also indicated a gradual fading of the effects over time. Furthermore, the findings suggested that younger women experienced greater and more sustained benefits compared to older patients.

In a study using Er:YAG, Ogrinc [15] reported an average of 2.54 necessary procedures to achieve continence. In his study, 77% reported significant improvement rates at 1-year follow-up, comparable to those of surgical interventions. Age was not an important factor in the outcome, with no differences between the pre- and post-menopausal women. Another interesting finding was that the symptoms of urinary incontinence showed improvement after just two interventions, with minimal additional effect after subsequent ones. On the other hand, Fistonic’s study [23], with a 72.3% improvement rate at 6 months, found better outcomes in patients under 39 years, as well as non-obese women, underlining the importance of early detection of low grades of SUI in women.

Gaspar published two studies [17,19] that reported subjective and objective clinical improvement in 69% and 50%, respectively, of the patients at the 6-month follow-up, as well as fading of the effect at the 18-month post-intervention mark, alleviated by maintenance interventions at 6 months and with improvement that can last for up to 3 years. Notably, he reported that, in cases with periurethral fibrosis due to childbirth-related injuries or previous surgeries, the effect of Er:YAG might be limited.

In Lin’s study [20], at 12-month follow-up, 62% of the patients were at least “satisfied” with the efficacy of the treatment. However, the symptom scores and objective cure rate were not sustained, compared to those at the 3-month follow-up. Most patients reported that the optimal effect was maintained for 3–6 months, underlining the possible necessity for a maintenance treatment 6 months after the initial one.

Two randomized control trials were assessed, one [21] comparing Er:YAG to PFMT and the other [24], to standard vaginal gel with estriol. The first study [21] found statistically significant improvements for the laser groups for up to 12 months, however the difference between groups was not significant at the end of the follow-up. The second one [24] reported statistically significant subjective improvement and suggested, at variance from Gaspar [46], that laser treatment effects are independent of any estrogen or non-hormonal pre-treatment when it comes to genitourinary syndrome of menopause that includes urinary incontinence.

Laser, radiofrequency, and infrared therapy have shown promising outcomes in clinical studies, with some of the cited studies including sufficiently large populations to support their conclusions. Limitations of this method are represented by multiple sessions often required to maintain results. In some cases, the benefits diminished over time, indicating the need for repeated treatments. Adverse effects were rare and generally mild, with the most commonly reported issues being a temporary warm sensation or pain and, in some instances, de novo urge incontinence. While the current findings are encouraging, longer-term follow-up studies are needed to evaluate the durability of these effects and establish standardized treatment protocols for optimal safety and efficacy.

### 3.3. Bulking Agents

Urethral bulking agents were initially introduced in the 1930s as a treatment for SUI, involving the use of paraffin to enhance urethral resistance. Since then, various agents have been introduced to the market, each exhibiting different safety, efficacy, and durability levels. Macroplastique, a polydimethylsiloxane elastomer, and Bulkamid, a polyacrylamide hydrogel, are the most used agents. Urolastic, PDMS-U, is a silicone gel that undergoes polymerization upon injection.

Two out of the 9 studies (see Supplementary Materials—Table S5) were retrospective, whereas the rest had a prospective design. One of the studies was the extension of a previously published 12-month follow-up intention-to-treat study. Bulkamid was the most commonly used bulking agent (927/1341), with Macroplastique being the second most commonly used (197/1341). In the single randomized controlled trial that we identified, Contigen, a discontinued collagen-based bulking agent, was administered to 102 individuals. One of the studies [34] did not provide information on the specific bulking agents that were utilized, choosing to report retrospectively on the success rates of previously injected materials.

Sokol [27] reported that, at the end of the 12-month follow-up, Bulkamid was non-inferior to Contigen; there were no statistically significant differences between the outcomes, with the two therapies showing similar efficacies in the >50% reduction in leakage and incontinence episodes, as well as the overall perception of individuals considering themselves cured or improved. Similarly, Maggiore [31] reported a subjective success rate of 74.4% at the 12-month follow-up, with statistically significant questionnaire results and a reduction in the number of leakage rates per 24h. Brosche [30] demonstrated Bulkamid’s durable outcomes at 7 years, suggesting favorable long-term safety profiles for such injections, as well as improvements in the quality of life in patients. The importance of its good safety profile was also insisted on by Pai et al. [33], who demonstrated that the proportion of patients experiencing improvement or cure of the symptoms at 3 months remained consistent during the entire follow-up period, with non-statistically significant decreases between 3 months and 5 years.

Ghoniem [28] released a follow-up to a previously published 12-month-follow-up intention-to-treat study in which Macroplastique was used. Of those considered dry at 12 months, 87% maintained cure at 24 months follow-up. Moreover, 41% of the cases that were considered improved at 12 months were considered dry at 24 months. Results from the I-QoL questionnaire showed statistically significant improvement from baseline. Zullo [29] reported an overall success rate of 77% after treatment with Macroplastique, comprising a 33% cure rate and 44% improvement rate. There were no statistically significant differences between 6 and 12 months’ rates. Carroll et al. [32] demonstrated that MPQ improved UDI-6 scores, quality of life questionnaire scores, and self-reported continence benefits, making Macroplastique a viable primary and secondary treatment for women experiencing SUI secondary to ISD, especially for those who have previously undergone extensive anti-incontinence treatments. Serati [35] discovered a significant correlation between surgeons’ skills and the absence of prior radical pelvic surgery, leading to success in 85 women who received Macroplastique injections and were monitored for 3 years. At the 3-year evaluation, 49% declared themselves cured, and 47% were objectively cured, with no statistically significant deterioration of objective cure rates over time. Overall, this bulking agent showed impressive, lasting results.

Studies with sufficiently large cohorts have supported the use of bulking agents for the treatment of stress urinary incontinence. One notable advantage of this treatment method is that it typically requires a single treatment session, which may enhance patient compliance. However, one study [33] reported the necessity of a booster injection to maintain efficacy. Overall success rates have varied between 40% and 83%, with most studies reporting an average success rate of approximately 45%. Despite these favorable outcomes, several limitations must be considered. The most commonly reported adverse events include urinary tract infections (UTIs) and urinary retention, with the latter potentially leading to prolonged catheterization or the need for additional interventions. Another important adverse event, voiding dysfunction, can manifest as difficulty initiating micturition, incomplete bladder emptying, or increased post-void residual volume, which can further contribute to recurrent infections or discomfort. Additionally, treatment availability can be limited by the reduced access to bulking agents across countries.

### 3.4. Stem Cell Therapy

Mesenchymal stem cells (MSCs) are multipotent cells that can differentiate into various cell types, including muscle and connective tissue cells. These cells have unique properties that make them attractive for regenerative medicine applications. MSCs have regenerative capabilities, and, when injected into the affected area, they promote tissue repair and regeneration. In the case of SUI, the hope is that MSCs can differentiate into muscle and connective tissue cells, strengthening the pelvic floor and improving continence. Moreover, they exhibit immunomodulatory properties, which can be beneficial in reducing inflammation and promoting a healing environment in the target tissue. MSCs exhibit the expression of distinct surface markers, such as CD105, CD73, and CD90. Conversely, they do not express CD45, CD34, CD14, CD11b, CD79a, CD19, and HLA class II, as assessed through flow cytometry. The mechanism can also include a peri-urethral bulking effect.

Seven studies were included in our survey (see Supplementary Materials—Table S6), of which four analyzed muscle-derived stem cells, two discussed adipose-derived stem cells, and the last compared mucosa-derived ones to surgical intervention.

Mahboubeh et al. [37] compared mucosa-derived stem cells to the minimally invasive mini-slings, finding that, while mini-slings showed better subjective results at 6 months, improvement rates were similar at 26 months, proving stem cells’ noninferiority. Moreover, the SC group had shorter intervention time and hospital stay and fewer complications.

Arjmand [36] showed statistically significant objective improvement rates between pre-intervention and 2 weeks after the intervention, as well as 6 and 24 weeks after the intervention, implying that there was a bulking effect, followed by a gradual decrease in its effect, and then a third regrowth of the stem cells. Subjectively, the findings were similar, with significant improvements between pre-op and 2-week scores, but not for the next two scores. Garcia-Arranz [38] reported that 5/10 of the patients had significant objective improvement after 3 months of follow-up, maintained until the end of the follow-up period of 12 months.

In regard to muscle-derived stem cells, Sharifiaghdas [39] reported a relapse of SUI at 24 months in five out of the ten clinically cured patients, as well as all of the partial responders. Gras [40] introduced a minced skeletal muscle tissue system, showing a positive correlation between biopsy weights and subjective and objective percentage improvement. He reported a 7–25% cure rate and a 57% improvement rate.

Stangel [41] reported first improvements observed at 4.7 months after transplantation, with further improvements until 8 months and sustained up to 2 years post-intervention; overall, 50% of the patients were considered continent, 25% had some improvement, and 25% had no benefits from the therapy. They also observed that longer exposure to estrogen leads to better results of stem cell therapy.

Lastly, Blaganje [42] combined myoblast with functional electrical stimulation for 5 weeks and discovered considerable improvements after a 2nd FES cycle and additional improvement at 3 and 6 months; at 6 months, 23% considered themselves cured, and 52% reported improvement. The discontinuation of FES did not negatively influence the results.

The use of stem cell therapy for managing SUI has shown promising results. No significant adverse reactions have been reported. Despite these positive findings, the widespread application of stem cell therapy remains limited due to its high cost and the technical complexity of the procedure, and, as such, the studies have included cohorts with a limited amount of participants, raising concerns about the generalizability of the findings. Furthermore, the high cost and technical challenges associated with stem cell isolation, preparation, and administration limit the widespread adoption of this method in clinical practice. As a result, its use is currently restricted to a select patient population. While the preliminary outcomes are encouraging, larger-scale studies are necessary to validate these results and determine the precise role of stem cell therapy in managing stress urinary incontinence.

## 4. Discussion

Stress urinary incontinence remains a prevalent issue for women, significantly impacting their quality of life and prompting social withdrawal. Many are often too ashamed to discuss with their medical care providers despite the negative effects on their emotional states, sexuality, and body image. Cases of younger women are also being reported in the literature with urinary symptomatology suggestive of stress urinary incontinence.

While modern therapies have slightly improved the situation for most affected individuals, there are still challenges in accurately diagnosing the pathology and establishing, along with the patient, the most effective therapeutic approach. As recommended by most societies, patients initially undergo pelvic floor muscle training along with medication, continence pessaries, and vaginal inserts [47,48,49,50]. However, inadequate adherence to behavior-based therapies, medication side effects, substantial expenses related to pessaries, and discomfort from the required insertion and removal of devices remain challenges [51,52]. When such therapies prove themselves to be ineffective or not sufficiently effective, minimally invasive interventions should be considered. The increasing demand for effective and minimally invasive approaches has led to the investigation of various injection techniques, as well as photobiomodulation therapy.

To be considered effective and safe for injection, an injectable agent must possess specific qualities, such as being nonantigenic, noncarcinogenic, not prone to dissociation or migration, and not causing severe inflammation. The selection of a natural and biocompatible material is crucial, in order to avoid interference with future surgical procedures. However, there have yet to be reported materials that can meet all criteria [53], and bulking agent therapy might have a lower cost-effective profile in comparison to some surgical techniques. However, the studies cited in this paper have shown >43% subjective and objective success rate up to 3 years after the procedure, with one study maintaining the reduction in symptoms noted at 3 months, up to 5 years. Mamut [54] emphasizes the need for proper patient selection and counseling regarding advantages and disadvantages, such as the necessity of repeat injections.

Stem cells, particularly mesenchymal stem cells, exhibit multipotent stromal properties, offering them the ability to differentiate into various tissues. Adipose tissue contains multipotent cells similar to MSCs that were initially harvested from bone marrow cells. However, cell therapy remains a demanding task due to its invasiveness and long and difficult preparation techniques. Moreover, a universally established protocol with a precise quantity of injected stem cells for achieving optimal outcomes is not currently in place. Arjmand [36] postulated that the suspension acts as a bulking agent in the first post-injection period, with considerable improvement that does not feel as satisfactory in the following weeks. Despite its perceived deterioration, the final results proved a significant improvement in the objective and subjective cure rates, most likely due to the proliferation, recruitment, and differentiation into functional contractile cells. In Garcia-Arranz’s study [38], a higher number of ASCs (40 million) showed better therapeutic results in mild to moderate incontinence. Alternatively, muscle-derived cells have shown a 50–59% complete response, at the end of their respective follow-up periods, but with a high recurrence of SUI. On the other hand, Stangel [41] used a lower number of infused cells but with a 50% success rate; the observed initial improvements were reported at an average of 4.7 months post-transplantation, continued until 8 months, and sustained up to 24 months. Despite the addition of functional electrical stimulation after the implantation of muscle-derived cells, at the 6-month follow-up, only 52% reported improvement; the discontinuation of the electrical stimulation did not influence the data. In comparison with surgical interventions, such as mini-slings, Mahboubeh [37] emphasized that the injection of stem cells was not inferior to MS, with shorter intervention time and fewer complications.

Platelet-rich plasma appears to be a promising, easily applicable, cost-effective viable therapeutic approach; compared to stem cells and bulking agents, acquiring it is simpler due to its autologous nature, and the preparation of the substance does not require extensive time. The proposed interval time between the 2–3 injections has been 4 to 6 weeks. All studies showed significant improvement rates at the final follow-up period, between 30 and 90%, compared to baseline. Behnia [13] also proved a 92% decrease in surgical procedure necessity and significant improvement that persisted through 2 years post-therapy. Another study by Pourebrahimi et al. [55] concluded that PRP injections show significant improvements in SUI symptoms, with shorter recovery times. Dankova et al. [56] suggested the need for clearer methodology insights in regard to optimal dosing, frequency, area of injection, and duration of treatment. However, PRP led to significant improvements in the patients’ stress urinary incontinence.

Laser techniques seem to require maintenance interventions. Er:YAG showed improvement rates of over 60% in all of the studies but with a fading effect at the 18-month mark. Radiofrequency treatment demonstrated different grades of improvement rate in half of the patients treated, including after 12 months. CO_2_ laser treatment, on the other hand, had disappointing results due to its temporary effectiveness, and showed no statistically significant improvement compared to the control groups. Laser treatments seem to have no long-term side effects, thus limiting the profile to minor immediate effects; however, more research should be carried out in order to establish the impact of the techniques. Another review conducted by Ranjbar et al. [57] concluded that the CO_2_ laser and Er:YAG laser are well-tolerated simple procedures that do not require prior extensive training.

The shortcoming of most conservative techniques seems to be represented by the temporary effects and the necessity of repeated treatments. Shah [58] proved that 100% of the women with more severe symptomatology are likely to choose surgical procedures, with a high rate of curing symptoms but longer hospital stay and a higher chance of complications, as opposed to nonsurgical treatments that would give them a variable chance of symptom improvement but pose a lower threat of complications or safety issues. Shah’s study contradicts the earlier paper by Robinson et al. [59], which showed that women would prefer fewer side effects, even if those implied less treatment efficacy.

Although this review is comprehensive, we acknowledge some limitations. While platelet-rich plasma (PRP), stem cell therapy, laser and radiofrequency treatments, and bulking agents have all demonstrated promising results in the management of stress urinary incontinence, each modality has significant limitations that should be taken into consideration. PRP has shown efficacy but requires repeated injections, is not universally available due to the necessity of preparation kits, and has been studied in small patient populations, limiting the generalizability of results. Similarly, stem cell therapy has yielded favorable outcomes with minimal adverse reactions; however, the high cost, technical complexity, and small study cohorts raise concerns about its accessibility and reproducibility. Laser and radiofrequency therapies have been supported by studies with sufficiently large cohorts, but their effects often require multiple treatment sessions, with some benefits fading over time. Bulking agents, offering the advantage of a one-time procedure in most cases, have demonstrated reproducible success rates, but their efficacy varies, and adverse events such as UTIs, urinary retention, and voiding dysfunction remain concerns. Some included studies were judged to be at high or moderate risk of bias, which may limit the certainty and interpretability of their findings. For example, the study conducted by Saraluck [9] demonstrated concerns with the randomization process (Domain 1), increasing the likelihood of selection bias and reducing internal validity. Inadequate randomization may compromise baseline comparability between groups and introduce confounding that cannot be reliably adjusted for.

Other studies (Alexander [16], Gambacciani [24], and Sokol [27]) were assessed as having “some concerns” related to missing outcome data and selective reporting. These methodological issues raise the risk of attrition and reporting biases, which can result in biased effect estimates or mask important findings. In non-randomized trials, moderate risks of bias were primarily associated with uncontrolled confounding (D1) and participant selection (D3), limiting causal inference. One study (Fistoníc [23]) was judged to be at serious risk of bias across multiple domains (D1, D3, D5), substantially affecting its internal validity.

These limitations may contribute to increased imprecision and heterogeneity in the overall evidence base, and caution is warranted when interpreting findings from studies at a higher risk of bias. However, the overall pattern of results among studies assessed as having low or moderate risk of bias demonstrates a degree of consistency in reported outcomes. This consistency strengthens confidence in the direction of the observed effects and supports the validity of the broader conclusions drawn in this review. Additionally, their availability is inconsistent across different regions. Substantial heterogeneity exists across the included studies, stemming from differences in study design, population characteristics, intervention protocols, outcome measures, and risk of bias. For instance, variations in laser parameters, treatment durations, and follow-up periods likely contributed to the differing magnitudes and durations of effect reported. Additionally, studies with a higher risk of bias often reported more favorable outcomes, raising concerns about potential overestimating treatment effects. This variability underscores the rationale for conducting the present review—not only to synthesize the existing evidence but also to qualitatively examine patterns in the data, identify methodological inconsistencies, and assess how these factors may influence reported outcomes.

To guide future research on the conservative treatment of stress urinary incontinence (SUI), we propose the adoption of a uniform set of outcome measures. Specifically, the pad weight test offers a quantifiable and reproducible method for assessing objective treatment efficacy. For subjective outcomes, we recommend the use of the Incontinence Quality of Life or the International Consultation on Incontinence questionnaires, which have been the most frequently employed tools across chosen studies and offer validated insight into patient-reported impact. While numerous outcome measures have been used in the literature, more does not necessarily mean better. Overall, while these therapies present valuable alternatives for managing stress urinary incontinence, their limitations underscore the need for larger-scale, high-quality randomized controlled trials, with standardized, validated outcome measures to improve treatment protocols, long-term efficacy, and accessibility for larger patient populations.

## 5. Conclusions

Surgical therapy remains a well-established option in the management of stress urinary incontinence; however, particularly for patients seeking less invasive approaches or those with contraindications to surgery, conservative and minimally invasive treatments could also be considered as first-line options. Nonsurgical therapies, including platelet-rich plasma (PRP), stem cell therapy, laser and radiofrequency treatments, and bulking agents, have shown efficacy not only in addressing stress urinary incontinence but also in managing the broader spectrum of the genitourinary syndrome of menopause.

Nevertheless, the strength of the evidence supporting these nonsurgical interventions is limited by methodological heterogeneity, moderate to high risk of bias, and short-term follow-up in some studies. Therefore, while current findings show promising results, definitive clinical recommendations should be made cautiously pending further high-quality randomized controlled trials.

Ultimately, the choice between surgical and nonsurgical interventions should be individualized, taking into account patient-specific factors such as symptom severity, treatment goals, age, the availability of procedures, and overall health status, alongside the strength and limitations of the existing evidence.

## Figures and Tables

**Table 1:** Number of identified clinical trials through the search of PubMed, Cochrane Library, and Clarivate Web of Science databases, based on keywords.

	Keywords Used	PubMed		Cochrane Library		Clarivate	
		Total	Clinical Trials Included	Total	Clinical Trials Included	Total	Clinical Trials Included
1	((platelet rich plasma) OR (PRP)) AND (stress urinary incontinence) AND (female)	15	5	14	4	12	5
2	((laser) OR (radiofrequency)) AND (stress urinary incontinence) AND (female)	172	13	14	5	113	10
3	(bulking agents) AND (stress urinary incontinence) AND (female)	260	9	41	8	192	9
4	(stem cell) AND (stress urinary incontinence) AND (female)	174	7	18	4	152	6

**Table 2:** Selected articles and their respective characteristics.

	Author	Year	Location	Time Frame	Study Design	Population	Mean Age	Therapy Used	Subjective	Objective
1	Saraluck et al. [9]	2023	Thailand	2022–2023	randomized, parallel two-group trial	60	30–68	A-PRP + PFMT vs. PFMT alone	ICIQ-FLUTS, IQOL-Q, PGI, subjective improvement	1 h PWT
2	Long et al. [10]	2021	Taiwan	2018	prospective interventional trial	20	44.5 ± 9.1	A-PRP	ICIQ-SF, UDI6, IIQ-7, OABSS, POPDI-6	1 h PWT
3	Athanasiou et al. [11]	2021	Greece		prospective observational trial	20	N/A	A-PRP	ICIQ-FLUTS, KHQ, PGI-I, VAS	1 h PWT
4	Grigoriadis et al. [12]	2024	Greece		double blind, randomized controlled trial	50	N/A	PRP vs. sham control group (sodium chloride 0.9%)	ICIQ-FLUTS, KHQ, PGI-I,	1 h PWT
5	Behnia-Willison et al. [13]	2020	Australia		prospective observational trial	62	55.98 ± 11.27	CO_2_ laser + PRP	APFQ	N/A
6	Temtanakitpaisan et al. [14]	2023	Thailand	2019–2021	randomized controlled trial	59	51.25 ± 11.36	CO_2_ vs. sham	ICIQ-SF	PF ultrasonography
7	Ogrinc et al. [15]	2015	Slovenia	2012–2013	prospective, non-randomized	175	49.7 (±10)	Er:YAG	ICIQ, ISI, VAS, satisfaction	N/A
8	Alexander et al. [16]	2022	Australia	2017–2020	participant-blinded, sham-controlled, parallel group	97	53 (34–79)	CO_2_ vs. sham	N/A	cough stress test, urodynamic stress incontinence, 24 h PWT
9	Gaspar et al. [17]	2022	Argentina		prospective noncontrolled	43	56 (33–64)	Er:YAG	ICIQ-SF	1 h PWT, 24 h PWT, 3 day voiding diary
10	Lauterbach et al. [18]	2022	Israel	2019–2020	double-blinded, prospective RCT	131	52 (±3.7)	CO_2_ vs. sham	UDI-6, ICIQ-UI	cough test, 1 h PWT
11	Gaspar et al. [19]	2017	Argentina			22	57.9 (33–66)	Er:YAG	ICIQUI-SF	1 h PWT
12	Lin et al. [20]	2017	Taiwan	2015	retrospective	30	52.6 ± 8.8	Er:YAG	ICIQ-SF, OABSS, UDI6, IIQ-7, POPDI-6, VAS	1 h PWT, urodynamic studies
13	da Fonseca et al. [21]	2023	Brazil		RCT	32	60.3 ± 8	Er:YAG vs. PFMT	KHQ, IQOL	1 h PWT
14	da Silva [22]	2023	Brazil		RCT	22	N/A	infrared + PFMT vs. placebo + PFMT	ICIQ-SF	biofeedback
15	Fistonic et al. [23]	2015	Croatia		prospective cohort	73	47 (41–54)	Er:YAG	ICIQ-SF, VAS	N/A
16	Gambacciani et al. [24]	2015	Italy		pilot prospective longitudinal	19/62	N/A	ER:YAG vs. standard vaginal gel with estriol	ICIQ-SF	N/A
17	Seki et al. [25]	2022	Brazil		3-arm double-blind RCT	114	50 ± 8.9	RF vs. LS vs. SHAM	IQOL, ICIQ-SF, Likert subjective scale, VAS	cough stress test, 1 h PWT, 7 day voiding diary
18	Elser et al. [26]	2010	USA		prospective open label	136	47 (26–87)	RF	IQOL, UDI-6, PGI-I	% of patients with a >50% reduction in SUI episodes, stress pad test
19	Sokol et al. [27]	2014	USA and Canada	2008–2011	single-masked, randomized, prospective, 2-arm, parallel	303	57.8	Bulkamid vs. Contigen (collagen)	ICIQ-UI, IQOL, Likert	% of patients with a >50% reduction in SUI episodes, stress pad test; at least 50% reduction from baseline in self-reported daily number if UI episodes; 24 h PWT, diary, responder rate
20	Ghoniem et al. [28]	2010	USA	2001–2004	extension of a previously published 12mo ITT study	67	62.4 ± 11.6	Macroplastique	IQOL, PGI-I	3 day voiding diary, cystoscopy, urodynamics, 1 h PWT, Stamey
21	Zullo et al. [29]	2010	USA	2005–2008	prospective cohort	27	77 (75–85)	Macroplastique	VAS	3 day voiding diary, stress test
22	Brosche et al. [30]	2021	Germany	2005-	retrospective	388	65.7 (±10.4)	Bulkamid	4 point scale (cured, improved, unchanged, worse), ICIQUI-SF, VAS	number of UI pads used, % of subjects requiring reinjection
23	Maggiore et al. [31]	2013	Italy	2008–2010	retrospective	82	54.3 ± 7.9	Bulkamid	ICIQSF, IIQ7, PGI-I	# of UI episodes in 24 h, 24 h PWT
24	Carroll et al. [32]	2019	USA	2011–2017	prospective	28/106	65.4 ± 8.3	Macroplastique	self-report, UDI-6, VAS QOL	3D ultrasound
25	Pai et al. [33]	2015	United Kingdom	2006–2011		256	N/A	Bulkamid	ICIQ, VAS	# of UI episodes in 24 h
26	Plotti et al. [34]	2018	Italy	1999–2013	retrospective	63	76 ± 8.2	UBAs	ICIQUI-SF, PGI-I, IIQ-7	N/A
27	Serati et al. [35]	2019	Italy	2008–2014	observational prospective	85	64 (40–76)	Macroplastique	ICIQ-SF, PGI-I, patient satisfaction scale, UDI	voiding diary, stress test
28	Arjmand et al. [36]	2017	Iran	2012	prospective	10	45.8 ± 8.7	abdominal subcutaneous adipose tissue	ICIQ	24 h voiding diary, 24 h PWT, urodynamic studies
29	Mahboubeh et al. [37]	2023	Iran	2016–2018	noninferiority randomized clinical trial	30	52	mucosa-derived SC vs. mini-sling	IIQ	Marshal
30	Garcia-Arranz et al. [38]	2020	Spain	2012–2014	prospective	10	56.8 ± 9	adipose-derived mesenchymal stem cells	SF36, ICIQUI-SF	urodynamic studies
31	Sharifiaghdas et al. [39]	2019	Iran	2013–2016	prospective	20	51.5 (30–70)	muscle-derived stem cells	IIQ-7, UDI-6	cough stress, 1 h PWT, urodynamic studies
32	Gräs et al. [40]	2014	Denmark	2010–2013	prospective	45	52 (34–80)	minced autologous skeletal muscle tissue	ICIQUI-SF	3 day voiding diary
33	Stangel-Wojcikiewicz et al. [41]	2014	Poland	2009–2011	prospective	16	56.75 ± 7.63	muscle-derived stem cells	Gaudenz	stress test, urodyn, PF-US
34	Blaganje et al. [42]	2012	Slovenia	2010	explorative clinical trial	38	52 (18–75)	autologous myoblast + functional electrical stimulation for 5 weeks	VAS, PGI-I, IQOL	3 day voiding diary, stress test, pad test, amount of leaked urine quantitatively

#: number; A-PRP: autologous platelet-rich plasma; APFQ: Australian Pelvic Floor Questionnaire; ICIQ-FLUTS: International Consultation on Incontinence Questionnaire—Female Lower Urinary Tract Symptoms; ICIQ-SF: International Consultation on Incontinence Questionnaire—Urinary Incontinence Short Form; IIQ-7: Incontinence Impact Questionnaire; IQOL-Q: Incontinence Quality of Life Questionnaire; KHQ: King’s Health Questionnaire; LS: laser; N/A: not available; OABSS: Overactive Bladder Symptom Score; PF: pelvic floor; PFMT: pelvic floor muscle training; PF-US: pelvic floor ultrasound; PGI-I: Patient Global Impression of Improvement; POPDI-6: Pelvic Organ Prolapse Distress Inventory 6; PWT: pad weight test; RCT: randomized controlled trial; RF: radiofrequency; SUI: stress urinary incontinence; UI: urinary incontinence; USA: United States of America; UDI-6: Urogenital Distress Inventory; VAS: visual analog scale; and YAG: yttrium-aluminum-garnet.

## Data Availability

No new data were created or analyzed in this study. Data sharing is not applicable to this article. The PROSPERO record can be downloaded from https://www.crd.york.ac.uk/PROSPERO/view/CRD420251011505, accessed on 14 March 2025. Risk of Bias tool RoB-2 can be accessed from https://methods.cochrane.org/risk-bias-2, accessed on 23 March 2024. Risk of Bias tool ROBINS-I can be accessed from https://methods.cochrane.org/robins-i, accessed on 23 March 2024.

## Supplementary Materials

The following supporting information can be downloaded at https://www.mdpi.com/article/10.3390/jcm14103268/s1, Table S1. Risk of bias assessment using RoB-2 tool for randomized controlled trials; Table S2. Risk of bias assessment using ROBINS-I tool for non-randomized trials; Table S3. Selected studies that included platelet-rich plasma therapy; Table S4. Selected studies that included laser, infrared and radiofrequency therapy; Table S5. Selected studies that included bulking agents; Table S6. Selected studies that included stem cells.

## Abbreviations

A-PRP	Autologous platelet-rich plasma
APFQ	Australian Pelvic Floor Questionnaire
BMI	Body Mass Index
CO_2_	Carbon Dioxide
Er:YAG	Erbium-doped yttrium aluminum garnet
FES	Functional electrical stimulation
ICIQ-FLUTS	International Consultation on Incontinence Questionnaire—Female Lower Urinary Tract Symptoms
ICIQ-SF	International Consultation on Incontinence Questionnaire—Urinary Incontinence Short Form
IIQ-7	Incontinence Impact Questionnaire
IQOL-Q	Incontinence Quality of Life Questionnaire
KHQ	King’s Health Questionnaire
LS	Laser
N/A	Not available
OABSS	Overactive Bladder Symptom Score
PF	Pelvic floor
PFMT	Pelvic floor muscle training
PF-US	pelvic floor ultrasound
PGI-I	Patient Global Impression of Improvement
POPDI-6	Pelvic Organ Prolapse Distress Inventory 6
PTG	Physical Therapy Group
PWT	Pad weight test
RCT	Randomized controlled trial
RF	Radiofrequency
SUI	Stress urinary incontinence
UI	Urinary incontinence
UDI-6	Urogenital Distress Inventory
UTI	Urinary tract infection
USA	United States of America
UUI	Urge Urinary Incontinence
VAS	Visual analog scale
VEL	Vaginal Erbium Laser
YAG	Yttrium-aluminum-garnet
