# Supplementary material for: Modern Conservative Management Strategies for Female Stress Urinary Incontinence: A Systematic Review
Source: J Clin Med. 2025 May 8;14(10):3268. doi: 10.3390/jcm14103268 (PMC12112232; doi:10.3390/jcm14103268)
Supplement: Supplementary file 1 [file jcm-14-03268-s001.zip › Suppl 1 - Table S1 Risk of bias assessment using RoB.pdf]

Table S1. Risk of bias assessment using RoB-2 tool for randomized controlled trials

Study	D1	D2	D3	D4	D5	Overall
Saraluck 2023	High	Low	Low	Low	Low	High
Grigoriadis 2024	Low	Low	Low	Low	Low	Low
Temtanakitpaisan 2023	Low	Low	Low	Low	Low	Low
Alexander 2022	Some concerns	Low	Low	Low	Low	Some concerns
Lauterbach 2022	Low	Low	Low	Low	Low	Low
da Fonseca 2023	Low	Low	Low	Low	Low	Low
da Silva 2023	Low	Low	Low	Low	Low	Low
Gambacciani 2015	Some concerns	Low	Some concerns	Low	Low	Some concerns
Seki 2022	Low	Low	Low	Low	Low	Low
Sokol 2014	Low	Low	Low	Low	Some concerns	Some concerns
Mahboubeh 2023	Low	Low	Low	Low	Low	Low

Domain 1: Risk of bias arising from the randomization process

Domain 2: Risk of bias due to deviations from the intended interventions

Domain 3: Missing outcome data

Domain 4: Risk of bias in the measurement of the outcome

Domain 5: Risk of bias in selection of the reported result
