# Supplementary material for: Modern Conservative Management Strategies for Female Stress Urinary Incontinence: A Systematic Review
Source: J Clin Med. 2025 May 8;14(10):3268. doi: 10.3390/jcm14103268 (PMC12112232; doi:10.3390/jcm14103268)
Supplement: Supplementary file 1 [file jcm-14-03268-s001.zip › Suppl 2 - Table S2 Risk of bias assessment using ROBINS.pdf]

Table S2. Risk of bias assessment using ROBINS-I tool for non-randomized trials

Study	D1	D2	D3	D4	D5	D6	D7	Overall
Long 2021	Moderate	Low	Low	Low	Moderate	Low	Low	Moderate
Athanasiou 2021	Moderate	Low	Low	Low	Low	Low	Low	Moderate
Behnia-Willison 2020	Low	Low	Low	Low	Low	Low	Low	Low
Ogrinc 2015	Low	Low	Low	Low	Low	Low	Low	Low
Gaspar 2022	Low	Low	Low	Low	Low	Low	Low	Low
Gaspar 2017	Low	Low	Low	Low	Low	Low	Low	Moderate
Lin 2017	Moderate	Low	Low	Low	Low	Low	Low	Moderate
Fistonic 2015	Moderate	Low	Moderate	Low	Moderate	Low	Low	Serious
Elser 2010	Moderate	Low	Low	Low	Moderate	Low	Low	Moderate
Ghoniem 2010	Moderate	Low	Low	Low	Low	Low	Low	Moderate
Zullo 2010	Low	Low	Low	Low	Low	Low	Low	Low
Brosche 2005	Moderate	Low	Low	Low	Low	Low	Low	Moderate
Maggiore 2013	Low	Low	Low	Low	Low	Low	Low	Low
Caroll 2019	Moderate	Low	Low	Low	Moderate	Low	Low	Moderate
Pai 2015	Low	Low	Low	Low	Low	Low	Low	Low
Plotti 2018	Low	Low	Moderate	Low	Moderate	Low	Low	Moderate
Serati 2019	Low	Low	Low	Low	Low	Low	Low	Low
Arjmand 2017	Moderate	Low	Low	Low	Low	Low	Low	Moderate
Garcia-Arranz 2020	Moderate	Low	Low	Low	Low	Low	Low	Moderate
Sharifiaghdas 2019	Low	Low	Low	Low	Low	Low	Low	Low
Gras 2014	Moderate	Low	Low	Low	Low	Low	Low	Moderate
Stangel-Wojcikiewicz 2014	Low	Low	Low	Low	Low	Low	Low	Low
Blaganje 2012	Moderate	Low	Low	Low	Low	Low	Low	Moderate

Domain 1: Risk of bias due to confounding

Domain 2: Risk of bias in classification of interventions

Domain 3: Risk of bias in the selection of participants into the study (or into the analysis)

Domain 4: Risk of bias due to deviations from intended interventions

Domain 5: Risk of bias due to missing data

Domain 6: Risk of bias arising from measurement of the outcome

Domain 7: Risk of bias in selection of the reported result
