# Supplementary material for: Modern Conservative Management Strategies for Female Stress Urinary Incontinence: A Systematic Review
Source: J Clin Med. 2025 May 8;14(10):3268. doi: 10.3390/jcm14103268 (PMC12112232; doi:10.3390/jcm14103268)
Supplement: Supplementary file 1 [file jcm-14-03268-s001.zip › Suppl 3 - Table S3 Selected studies that included platelet.pdf]

Table S3. Selected studies that included platelet-rich plasma therapy

	Author	Therapy used	Intervention period	Amount	Platelet concentration	Adverse events	Results
1	Saraluck et al.[9]	A-PRP+PFMT vs. PFMT alone	2 injections, 1 month interval	5ml A-PRP	×1.6 (Regen)	none	90% reported a >50% improvement in the A-PRP+PFMT group, vs. 14% in the PFMT alone group
2	Long et al.[10]	A-PRP	3 injections, 1 month interval	5ml A-PRP	×1.6 (Regen)	none	significant and lasting effectiveness; POPDI-6 showed improved scores, but not statistically significant; younger women might have better treatment outcome
3	Athanasiou et al.[11]	A-PRP	2 injections, 4-6 weeks interval	N/A	N/A	N/A	statistically significant improvement at 3 months after treatment, further improvement at 6 months; mean reduction of 50.2% in urine loss at 1 hour pad weight test; at 6 months: 80% reported to be "at least improved"
4	Grigoriadis et al.[12]	PRP vs. sham control group (sodium chloride 0.9%)	2 injections, 4-6 weeks interval	N/A	N/A	N/A	significant decrease in the PRP group vs. sham group; subjective cure was statistically significantly higher in the PRP group; significant 1 hour pad weight test improvement at 6 months for the PRP group
5	Behnia-Willison et al.[13]	CO_2 laser + PRP	3 treatments, 4-6 weeks interval	N/A	×1.6 (Regen)	none	66% reported improvement from T1 to T2; at T3, 62% improvement of symptoms; from T1 to T2, all bladder function variables were improved significantly; at T3, significant improvements were maintained for all bladder function variables, except pad usage

A-PRP: autologous platelet rich plasma; PFMT: pelvic floor muscle training
