# Supplementary material for: Modern Conservative Management Strategies for Female Stress Urinary Incontinence: A Systematic Review
Source: J Clin Med. 2025 May 8;14(10):3268. doi: 10.3390/jcm14103268 (PMC12112232; doi:10.3390/jcm14103268)
Supplement: Supplementary file 1 [file jcm-14-03268-s001.zip › Suppl 4 - Table S4 Selected studies that included laser.pdf]

Table S4. Selected studies that included laser, infrared and radiofrequency therapy

	Author	Therapy used	Intervention period	Adverse events	Results
1	Temtanakitpaisan et al.[14]	CO_2 vs. sham	Four times, 28-35 days apart	minimal vaginal pain during the procedure	statistically significant improvement in both groups, but no differences between groups; no changes in bladder neck descent or levator hiatal area immediately after intervention or 3 months after
2	Ogrinc et al.[15]	Er:YAG	2nd procedure after 4-6 weeks, 3rd procedure 6 months after the 1st	mild discomfort/pain during the procedure	transient UUI after the first procedure in 11 patients (6.3%)<br>after the second procedure, de novo UUI in seven patients (4%) from the SUI group.<br>significant improvement in 77% of patients
3	Alexander et al.[16]	CO_2 vs. sham	Three times, 4 weeks apart	vaginal bleeding (3 patients who had undergone laser procedure, 1 in the sham group)	no differences reported at 3 months post-procedure between sham and active treatment; patient reported outcomes and health-related QoL were comparable
4	Gaspar et al.[17]	Er:YAG	Three times, 20 day interval; a single maintenance session at 18 months follow-up	warm sensation during treatment; 9% reported de novo urge urinary incontinence (resolved within 7 days)	significant improvement in all outcome measures assessed at all follow-up appointments<br>18months follow-up: a fading of the effect, alleviated by maintenance at 6months
5	Lauterbach et al.[18]	CO_2 vs. sham	One maintenance intervention	warm sensation during treatment (92% of the study group)	statistically significant improvements at 3 months in objective and subjective measurements; at 6 months, positive cough test, 1 hour PWT +

					3 questionnaires were similar in the study and control groups
6	Gaspar et al.[19]	Er:YAG	Two times, 3 weeks apart	1 patient pelvic pain, 2 patients dysuria (disappeared within 24h)	statistically significant reduction of quality of life questionnaire score and PWT, maintained up to 6 months<br>subjective: 3 months after the procedure: 64% cured,1 8% improved; 6months: 46% cured, 23% improved.<br>objective: 3 months: 82%, 6months: 50%; slight improvement in severity
7	Lin et al.[20]	Er:YAG	Two times, 4 weeks apart	itching, discharge, discoloration, bleeding, pain or burning sensation; no major adverse effects	12 months after completing two sessions: 2 (6.8%) patients were very satisfied with the efficacy, 16 (55.2%) patients were satisfied, 8 (26%) remained unchanged, and 4 (13.8%) patients were dissatisfied; symptoms were better at 3 months but not sustained to 12 months
8	da Fonseca et al.[21]	Er:YAG vs. PFMT	ER:YAG: three times, 1 month apart; PFMT: twice a week, 3 months	N/A	Laser group achieved improvement at all consultations, but PFMT group achieved improvement only at 1, 3, 6 months. KHQ showed no difference between groups;<br>Laser group: greater reduction in urinary loss, as measured using the PWT, at 6 months and 12 months after treatment; no difference vs. PFMT at the end of the follow-up.
9	da Silva [22]	infrared + PFMT vs.	1 laser interventi on,	None	The muscular strength increased significantly for the infrared+PFMT

		placebo + PFMT	followed by 10 sessions, twice a week, of PFMT		group, where the majority of patients gained more than twice the strength in the pelvic apparatus
10	Fistonic et al.[23]	Er:YAG		warmth/pricking/irritation during treatment, discharge in the next few days; slight edema that disappeared within 48hours; de novo UUI that disappeared after 8days	better results in women with normal BMI, as well as women younger than 39 years old
11	Gambacciani et al.[24]	ER:YAG (VEL) vs. standard vaginal gel with estriol	Three times, 30 days apart	N/A	VEL group showed statistically significant improvement, up to the 24th week of observation
12	Seki et al.[25]	Radiofrequency vs. laser vs. sham	Three times, 1 month apart	N/A	subjective improvement and objective cure: 72.6% and 45.2% in LS and in 61.7% and 44.7% in RF, both significantly higher than the 30.0% and 14.0% in control group; Improvement in QoL was also verified by the I-QoL and ICIQ-SF in favor of the first two groups
13	Elser et al.[26]	RF	One time	dysuria 5.2%, urinary retention 4.4%, pain 2.9%, UTI 2.9%	results at 18 months post-procedure reveal consistent, statistically significant improvements; nearly half of them are significant durable reductions in activity related leaks and QoL improvement

BMI: Body Mass Index; Er:YAG: Erbium-doped Yttrium Aluminium Garnet; KHQ: King's Health Questionnaire; LS: Laser; N/A: Not Available; PFMT: Pelvic Floor Muscle Training; PTG: Physical Therapy Group; PWT: Pad Weight Test; QoL: Quality of Life; RF: Radiofrequency; SUI: Stress Urinary Incontinence; UUI: Urge Urinary Incontinence; VEL: Vaginal Erbium Laser
