# Supplementary material for: Modern Conservative Management Strategies for Female Stress Urinary Incontinence: A Systematic Review
Source: J Clin Med. 2025 May 8;14(10):3268. doi: 10.3390/jcm14103268 (PMC12112232; doi:10.3390/jcm14103268)
Supplement: Supplementary file 1 [file jcm-14-03268-s001.zip › Suppl 5 - Table S5 Selected studies that included bulking agents.pdf]

Table S5. Selected studies that included bulking agents

	Author	Therapy used	Intervention period	Adverse events	Results
1	Sokol et al.[27]	Bulkamid vs. Contigen (collagen)	once	UTI, transient pain, acute retention	>50% reduction in leakage and incontinence episodes: 53.2% Bulkamid (B), 55.4% Contigen (C) at 12mo; 47.2% B and 50% C reported 0 SUI episodes; 77.1% B and 70% C considered themselves cured of improved; B is not inferior to C
2	Ghoniem et al.[28]	Macroplastique	once	UTI, transient hematuria, dysuria, overactive bladder, itching, yeast infection, hysterectomy	at 24 months, 84% had retained success since 12 months (67% were dry). Of the dry patients at 12 months, 87% maintained cure at 24 months. 41% of those considered improved at 12 months were dry at 24 months. IQOL showed SS improvement from baseline.
3	Zullo et al.[29]	Macroplastique	once	none	after 12 months, 44% objective cure rate; 33% improvement rate, 22% failure rate; overall success rate 77%; no statistically significant difference
4	Brosche et al.[30]	Bulkamid	once	urinary retention, UTI, hematuria, worsened symptoms	65.2% felt cured or improved (16% cured, 49.2% improved); 2.3% worsened, 19.1% received other therapy
5	Maggiore et al.[31]	Bulkamid	once	UTI, pain, hematuria, de novo urgency	subjective success rate 74.4% at 1 year follow-up; IIQ7 significantly improved; mean number of episodes of urine leakage/24h decreased significantly
6	Carroll et al.[32]	Macroplastique	once	none	1 patient achieved complete dryness; success rate of 37% after first MPQ injection, with a 41% success rate after final

7	Pai et al.[33]	Bulkamid	Once; 18 patients required a booster	de novo overactivity, cystitis, urinary retention,	82.8% had a subjective improvement (subjectively cured or significant improvement) at 3 month follow up. 42.9% of the patients were cured. The percentage of patients cured/improved at 3 months was maintained throughout follow up with a non-statistically significant reduction between 3 months-5 years. The median ICIQ score showed a significant improvement at 3 months follow-up. This improvement was maintained at 12 months and on subsequent yearly follow up to 5 years
8	Plotti et al.[34]	Urethral bulking agents	once	N/A	24% patients were considered cured; 19% improvement rate; 57% failure rate; overall success of 43%
9	Serati et al.[35]	Macroplastique	once	de novo bladder overactivity, voiding dysfunction, pain	At 3 years after intervention, 49% declared themselves cured. At the 3-year evaluation, 47% were objectively cured. There was no significant deterioration of objective cure rates over time

B: Bulkamid; C: Contigen; ICIQ: International Consultation on Incontinence Questionnaire; IIQ7: Incontinence Impact Questionnaire; I-QOL: Incontinence Quality of Life Questionnaire; MPQ: Macroplastique; SS: Statistically Significant; SUI: Stress Urinary Incontinence; UTI: Urinary Tract Infection
