# Supplementary material for: Modern Conservative Management Strategies for Female Stress Urinary Incontinence: A Systematic Review
Source: J Clin Med. 2025 May 8;14(10):3268. doi: 10.3390/jcm14103268 (PMC12112232; doi:10.3390/jcm14103268)
Supplement: Supplementary file 1 [file jcm-14-03268-s001.zip › Suppl 6 - Table S6 Selected studies that included stem cells.pdf]

Table S6. Selected studies that included stem cells

	Author	Therapy used	Intervention period	Amount	Adverse events	Results
1	Arjmand et al.[36]	abdominal subcutaneous adipose tissue	1x	1,180,000 cells/ml, 10 ml	voiding difficulty	statistically significant differences preoperatively and after 2 weeks, as well as between 6 and 24 weeks, but not between 2 and 7 weeks
2	Mahboubeh et al.[37]	mucosa-derived stem cells vs. mini-sling	1x		higher rate of dyspareunia in MS	mucosa-derived stem cells treatment is not inferior to mini sling; shorter intervention time, and fewer complications
3	Garcia-Arranz et al.[38]	adipose-derived mesenchymal stem cells	1x	40 x 10^6 cells, 2ml	none	5 women reported objective improvement of >50%; subjective improvement of 70-80% from baseline
4	Sharifiaghdas et al.[39]	muscle-derived stem cells	1x	50×10^6 cells	none	12 months follow-up: 59% complete response, 12% partial response, 29%, failed; at the end of 2-year follow-up, recurrence of symptoms in 5/10 cured patients as well as all the partial responders
5	Gräs et al.[40]	minced autologous skeletal muscle tissue	1x	N/A	Urinary tract infection, pain, infection, hematoma	significant reduction of the mean number of stress leaks and the impact of symptoms; 10/16 patients with uncomplicated SUI improved, 5/16 had no improvement, and 1/16 had a slight worsening in leaks; 8/15 patients with complicated SUI improved, 6/15 had no improvement
6	Stangel-Wojcikiewicz et al.[41]	muscle derived stem cells	1x	0.6-25 x 10^6	none	50% achieved continence, 25% some improvement and 25% no improvement; first improvements were

						reported 4.7 months after transplantation, continued improvements until 8 months, and sustained up to 24 months
7	Blaganje et al.[42]	autologous myoblast + functional electrical stimulation for 5 weeks	1x	1-50 x 10^6 (2ml)	cystitis, tenderness	6 weeks after implantation, after a 2nd functional electrical stimulation cycle - considerable improvement; additional improvement at 3 and 6 months; 23% cured, 52% reported improvement at 6 months

SUI: stress urinary incontinence
